# Anti-Inflammatory Effects of Ellagic Acid on Keratinocytes via MAPK and STAT Pathways

**DOI:** 10.3390/ijms22031277

**Published:** 2021-01-28

**Authors:** Tae-Young Gil, Chul-Hee Hong, Hyo-Jin An

**Affiliations:** 1 Department of Pharmacology, College of Korean Medicine, Sangji University, Wonju-si 26339, Gangwon-do, Korea; sophia14t@gmail.com; 2 Department of Korean Meidicne Ophthalmology & Otolaryngology & Dermatology, College of Korean Medicine, Sangji University, Wonju-si 26339, Gangwon-do, Korea; hong7250@sangji.ac.kr

**Keywords:** atopic dermatitis, inflammation, chronic disease, ellagic acid, MAPKs, STATs

## Abstract

Atopic dermatitis (AD) is a chronic inflammatory skin disease that is characterized by an impaired skin barrier and intense itchiness, which decreases the individual’s quality of life. No fully effective therapeutic agents have prevailed for AD due to an insufficient grasp of the complex etiology. Ellagic acid (EA), a natural compound, has anti-inflammatory properties in chronic diseases. The effects of EA on AD have not yet been explored. The present study investigated the effects of EA on TNF-α/IFN-γ-stimulated HaCaT keratinocytes and house dust mite-induced AD-like skin lesions in NC/Nga mice. Treatment with EA suppressed inflammatory responses in keratinocytes by regulating critical inflammatory signaling pathways, such as mitogen-activated protein kinases and signal transducers and activators of transcription. In vivo studies using a DfE-induced AD mouse model showed the effects of EA administration through ameliorated skin lesions via decremented histological inflammatory reactions. These results suggest that EA could be a potential therapeutic alternative for the treatment of AD by inhibiting inflammatory signaling pathways.

## 1. Introduction

Atopic dermatitis (AD) is a chronic inflammatory skin disease accompanied by a multifactorial interplay between innate and adaptive immune responses [1]. It is characterized by an impaired skin barrier, skin lesions, and intense pruritus, which lower the individual’s quality of life [2]. As the most common inflammatory skin disease, AD affects 15–30% of children and almost 14.3% of adults [3]. The high incidence of AD causes substantial social and financial burdens on society [4]. Since the etiology of AD is complex, verified approaches to managing AD involve Janus kinase (JAK) inhibitors, corticosteroids, or regulators of the immune system such as dupilumab [5,6,7]. The critical common basis of these treatments is controlling inflammation.

An inflammatory response is shown in the impaired skin barrier and skin plays a crucial role as a protective barrier against not only the loss of moisture but also the entry of toxic or infectious factors [8]. Keratinocytes, the majority of epidermal cells, contribute to the pathogenesis of inflammatory skin diseases, including AD [9]. Keratinocytes are affected by different factors, such as environmental allergens, scratching of the area, and bacterial superimposition, leading to the production of inflammatory cytokines [10]. Keratinocytes and Langerhans cells are induced to secrete pro-inflammatory cytokines, including interleukin (IL)-6, tumor necrosis factor-alpha (TNF-α), and thymic stromal lymphopoietin (TSLP) [11]. TSLP, a crucial regulator of Th2-driven inflammation, is increased in skin lesions, leading T cell migration [12]. Activated keratinocytes produce chemokines, such as regulated on activation, normal T-cell expressed and secreted (RANTES/CCL5), thymus and activation-regulated chemokine (TARC/CCL17), and macrophage-derived chemokine (MDC/CCL22) [13]. These T helper type 2 (Th2)-related chemokines lead to the augmented infiltration of Th2 cells into inflammatory tissues [14]. Keratinocytes stimulated by a TNF-α and IFN-γ mixture (TNF-α/IFN-γ) can activate various signaling pathways, such as nuclear factor-kappa B, mitogen-activated protein kinases (MAPKs), and signal transducer and activator of transcription (STAT) pathways [15].

Ellagic acid (EA) is a natural phenolic compound found in a variety of fruits and vegetables [16]. EA has strong antioxidant and anticancer properties owing to its antiproliferative and apoptotic effects [17,18,19]. EA has anti-inflammatory effects in acute or chronic models of ulcerative colitis [20], Crohn’s disease [21], and rheumatoid arthritis [22]. Recently, an increasing number of studies have investigated EA as a potential protective agent of the liver or skin via cell proliferation, apoptosis, DNA damage, angiogenesis, and inflammatory properties [23]. Although previous studies have investigated the anti-inflammatory effects of EA on skin disease, the effects on AD-like skin lesions and underlying molecular mechanisms have not, to our knowledge, previously been considered.

In this study, we investigated the influence of EA and the available molecular plant mechanism in HaCaT immortalized human keratinocytes and NC/Nga mice. Using HaCaT cells, we studied the effects of EA on inflammation and tried to elucidate the underlying molecular mechanism, i.e., the MAPK and STAT signaling pathways.

## 2. Results

### 2.1. Effects of EA on Inflammatory Cytokines and Chemokines in TNF-α/IFN-γ-Stimulated HaCaT Keratinocytes

**Figure 1:**
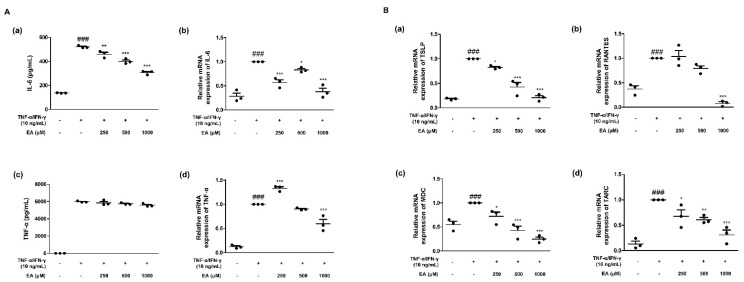
Effects of ellagic acid (EA) on inflammatory cytokines and chemokines in HaCaT keratinocytes. (**A**) Production of inflammatory cytokines and their mRNA expression were determined. Production of (**a**) interleukin-6 (IL-6) and (**c**) tumour necrosis factor-α (TNF-α) was determined by enzyme-linked immunosorbent assay (ELISA). Cells were treated with 250, 500, or 1000 μM of EA for 1 h prior to the addition of TNF-α/IFN-γ, and cells were incubated for 24 h. mRNA expression of (**b**) IL-6 and (**d**) TNF-α was assayed by qRT-PCR. (**B**) mRNA expression of pro-inflammatory chemokines. mRNA expression of (**a**) thymic stromal lymphopoietin (TSLP), (**b**) normal T-cell expressed and secreted chemokine (RANTES), (**c**) macrophage-derived chemokine (MDC), and (**d**) thymus and activation-regulated chemokine (TARC) was assayed by qRT-PCR. Cells were treated with 250, 500, or 1000 μM of EA for 1 h prior to the addition of TNF-α/IFN-γ, and cells were incubated for 6 h. Then, total RNA was isolated from HaCaT keratinocytes, and qRT-PCR was performed. Data are presented as the mean ± standard error mean (SEM) of three independent experiments (*n* = 3); * *p* < 0.05, ** *p* < 0.01 and *** *p* < 0.001 versus the only TNF-α/IFN-γ-treated group, ^###^ *p* < 0.001 versus the control group by ANOVA and Dunnett’s post-hoc test.

Epidermal keratinocytes play various roles in immune responses related to allergic dermatitis and other skin diseases [24]. To investigate whether EA suppresses the expression of TNF-α/IFN-γ-induced inflammatory cytokines and chemokines, we performed enzyme-linked immunosorbent assays (ELISA) and quantitative reverse transcription-polymerase chain reaction (qRT-PCR) assays. TNF-α and IL-6 are prominent cytokines in the pathogenesis of skin disorders [25]. We examined the production of inflammatory mediators and their mRNA expression (Figure 1A). EA inhibited the increased production caused by TNF-α/IFN-γ stimulation. EA treatment at 1000 μM significantly (*** *p* < 0.001) suppressed the production and mRNA expression of inflammatory cytokines. In addition, human TSLP activates CD11c (+) dendritic cells and leads to the production of Th2-attracting chemokines, including TARC, MDC, and RANTES [26,27]. Figure 1B shows the effects of EA on the mRNA expression of Th2-related chemokines in TNF-α/IFN-γ-stimulated HaCaT keratinocytes. TNF-α/IFN-γ increased the mRNA expression of the chemokines (a) TSLP, (b) RANTES, (c) MDC, and (d) TARC, as shown in Figure 1B. EA suppressed the expression of these chemokines.

### 2.2. Effects of EA on Inflammatory Signaling Pathways in TNF-α/IFN-γ-Stimulated HaCaT Keratinocytes

**Figure 2:**
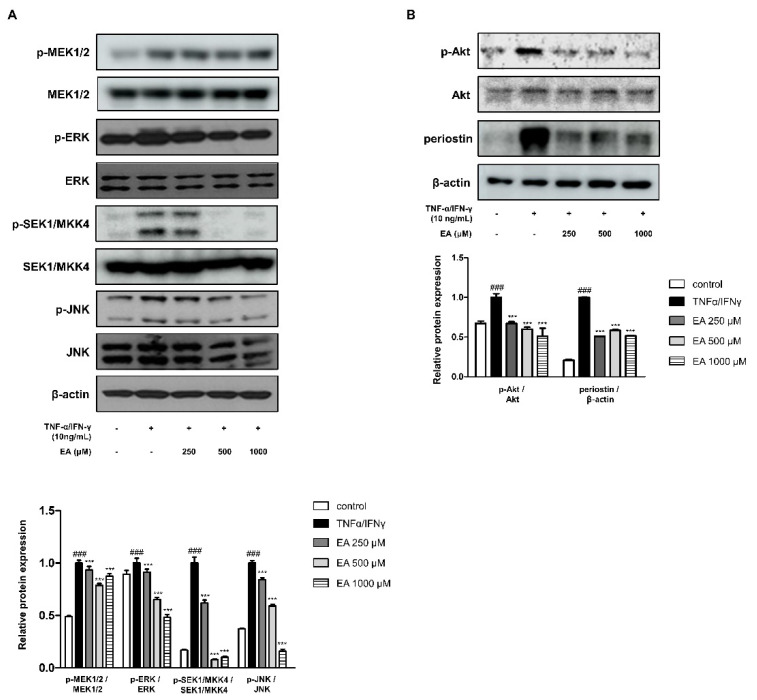
Effects of EA on mitogen-activated protein kinase (MAPK)-related signaling pathways in HaCaT keratinocytes. Cells were treated with 250, 500, or 1000 μM of EA for 1 h prior to the addition of TNF-α/IFN-γ and incubated for 10–20 min. Phosphorylation of (**A**) MEK1/2-ERK and SEK1/MKK4-JNK cascades was assayed by western blot analysis. The graph shows the ratio of phosphorylated MEK1/2-ERK and SEK1/MKK4-JNK to total MEK1/2-ERK and SEK1/MKK4-JNK, respectively. (**B**) Phosphorylation of Akt and periostin was analyzed by western blot assay. The ratios of p-Akt to Akt and periostin to β-actin are presented. Values represent the mean ± standard error mean (SEM) of triplicate independent experiments (*n* = 3). *** *p* < 0.001 versus the only TNF-α/IFN-γ-treated group, ^###^ *p* < 0.001 versus the control group by ANOVA and Dunnett’s post-hoc test.

MAPKs are crucial inflammatory cytokine regulators in keratinocytes [28], and there are three well-known subfamilies of MAPKs in the mammalian system, including extracellular signal-regulated kinases (ERKs), c-Jun N-terminal kinases (JNKs), and p38 MAPKs [29]. MAPK kinases (MAPKKs), in turn, activate MAPKs through serial phosphorylation [30]. Activation of MAPKKs and phosphorylation of MAPKs was determined in HaCaT cells using western blot analysis. As shown in Figure 2A, cells treated with TNF-α/IFN-γ activated MEK1/2-ERK and SEK1/MKK4-JNK. EA treatment at 1000 μM significantly suppressed p-MEK1/2 and p-ERK expression. EA showed inhibitory effects on p-SEK1/MKK4 and p-JNK in a concentration-dependent manner. Meanwhile, another inflammatory signaling pathway, phosphoinositide 3-kinase/Akt, activates protein kinase C, MAPK, and nuclear factor κB [31]. Figure 2B shows other inflammation-related protein expression. Phosphorylation of Akt in keratinocytes treated with TNF-α/IFN-γ was suppressed by EA treatment. Additionally, augmented protein expression of periostin in TNF-α/IFN-γ-treated cells was inhibited by EA (Figure 2B). Periostin is profoundly involved in the etiology of AD and many inflammatory skin diseases [32].

### 2.3. Effects of EA on the JAK/STAT Signaling Pathway in TNF-α/IFN-γ-Stimulated HaCaT Keratinocytes

**Figure 3:**
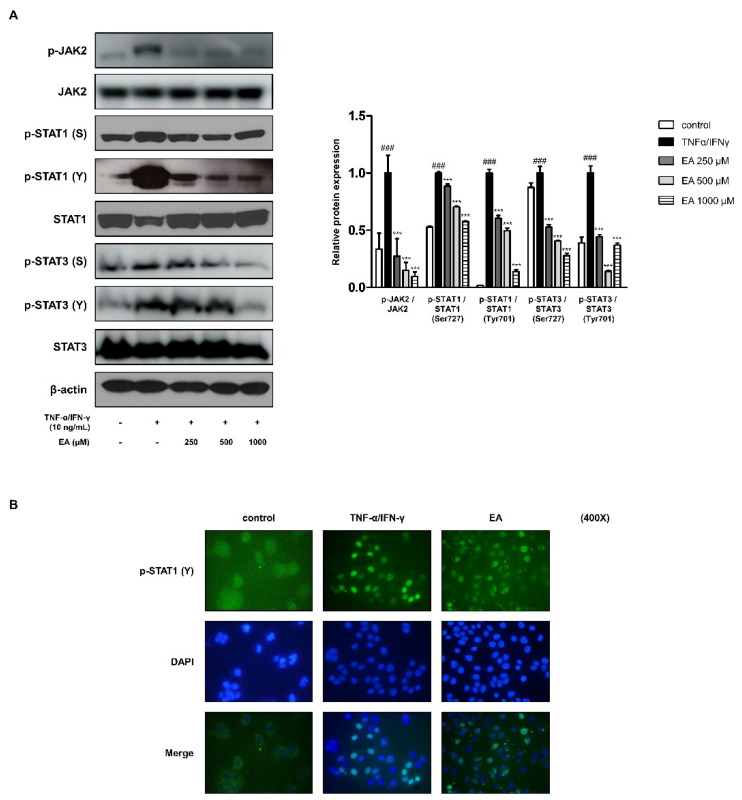
Effects of EA on Janus kinase (JAK)/STAT signaling pathways in activated HaCaT keratinocytes. Cells were treated with 250, 500, or 1000 μM of EA for 1 h prior to the addition of TNF-α/IFN-γ and were incubated for 1 h. (**A**) Phosphorylation of JAK2, STAT1, and STAT3 was determined by western blot assay. The graph shows the ratio of phosphorylated JAK2, STAT1, and STAT3 to total JAK2, STAT1, and STAT3, respectively. (**B**) Translocation of phosphorylated STAT1 (Y) was visualized by immunofluorescence. The nuclei were counterstained with DAPI (blue). The stained cells were visualized with a fluorescence microscope at 400× magnification. Values represent the mean ± standard error mean (SEM) of triplicate independent experiments (*n* = 3). *** *p* < 0.001 versus the only TNF-α/IFN-γ-treated group, ^###^ *p* < 0.001 versus the control group by ANOVA and Dunnett’s post-hoc test.

The Janus kinase (JAK)/STAT signaling pathway for skin inflammation is well understood in humans carrying mutations in genes encoding JAK or STAT proteins [33]. This signaling pathway is essentially involved in the pro-inflammatory action of many cytokines in AD [34]. Phosphorylated STAT1 and STAT3 on serine and tyrosine by TNF-α/IFN-γ-stimulation using western blot analysis is shown in Figure 3A. This is caused by JAK2 activation, which phosphorylates and activates STATs. EA inhibited the phosphorylation of JAK2, STAT1, and STAT3. Ligand activation of JAKs induces the phosphorylation of the receptor chains where the STATs bind. In succession, STATs are phosphorylated on tyrosine, dimerize, translocate to the nucleus, and activate transcription [35]. We studied the translocation of phosphorylated STAT1 on tyrosine using immunofluorescence analysis (Figure 3B). The translocation of phosphorylated STAT1 on tyrosine701 (Y) was detected in keratinocytes (green). The nuclei were counterstained blue with 4′,6-diamidino–2–phenylindole (DAPI). EA treatment at 1000 μM inhibited pSTAT1(Y) nuclear translocation as compared to only TNF-α/IFN-γ-stimulated cells.

### 2.4. Effects of EA on Dermatophagoides Farinae Crude Extract (DfE)-Induced AD-Like Skin Lesions in NC/Nga Mice

**Figure 4:**
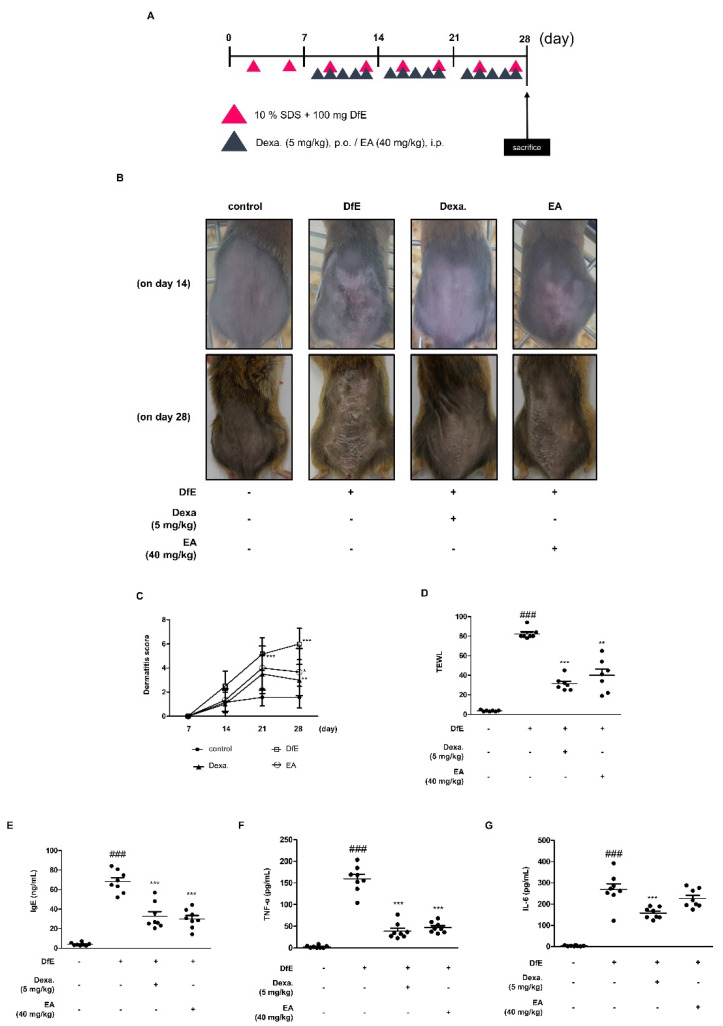
Effects of EA on DfE-induced AD-like skin lesions in NC/Nga mice. (**A**) Experimental scheme for the induction of AD and treatment of EA in NC/Nga mice. (**B**) Comparison of DfE-induced dermatitis in NC/Nga mice after EA (40 mg/kg/day) intraperitoneal administration. Representative photographs of dorsal regions of the mice from each group 14 and 28 days after AD induction. (**C**) Dermatitis scores over 4 weeks. The dermatitis scores were determined as the sum of scores graded as 0 (none), 1 (mild), 2 (moderate), or 3 (severe) for each of the four symptoms; erythema/hemorrhage, scarring/dryness, edema, and excoriation/erosion. (**D**) Transepidermal water loss (TEWL) was measured with a gpskin barrier light on day 28 after AD induction. (*n* = 7) (**E**) Serum IgE, (**F**) serum TNF-α, and (**G**) serum IL-6 levels were measured by ELISA (*n* = 8). Data are expressed as the mean ± standard deviation (SD). * *p* < 0.05, ** *p* < 0.01 and *** *p* < 0.001 versus the only DfE-treated group, ^###^ *p* < 0.001 versus the control group by ANOVA and Dunnett’s post-hoc test.

To investigate the effect of EA on AD-like symptoms, we evaluated the effects of EA on a DfE-induced AD animal model. DfE is one of the principal sources of dust mite allergens that cause allergic diseases [36]. Clinical features, including dorsal skin condition, dermatitis score, and transepidermal water loss (TEWL) were analyzed (Figure 4). We induced AD-like skin lesions by DfE for 4 weeks. EA treatment (40 mg/kg) and positive control dexamethasone (Dexa.) treatment (5 mg/kg) were administered for 3 weeks, followed by DfE administration for 1 week (Figure 4A). We found that in the AD-like skin lesions in the DfE-induced group on day 14 and 28, the severity had increased in the group comparing to the Dexa. or EA treatment groups (Figure 4B). On day 28, the final day of the experiment, AD-like dorsal lesions of the EA group exhibited amelioration as compared to the DfE group. For the 4-week experimental period, we estimated the condition using the symptom score [37] and found significant improvement in the final week (Figure 4C). One of the representative details of AD is the damaged skin barrier, which is accompanied by dry skin and augmented TEWL [38]. We found decreased TEWL by EA treatment under the DfE-induced AD-like lesion (Figure 4D). Besides the external features of AD, such as dryness, we investigated the internal features in serum. Serum immunoglobulin (Ig)E levels are extremely elevated in patients with AD and are considered an AD biomarker [39]. We evaluated the production of serum IgE after euthanizing the mice. Increased IgE production was found in the DfE-induced AD mouse models (Figure 4E). This effect was significantly inhibited by treatment with Dexa. and EA. Also, serum pro-inflammatory cytokines, TNF-α and IL-6 were measured in the DfE-induced AD animal model (Figure 4F,G). As the representative pro-inflammatory markers, TNF-α and IL-6 were reduced by the EA, indicating the anti-inflammatory effects.

### 2.5. Effects of EA on Skin Integrity and Mast Cell Infiltration in DfE-Induced AD-Like NC/Nga Mouse Skin Lesions

**Figure 5:**
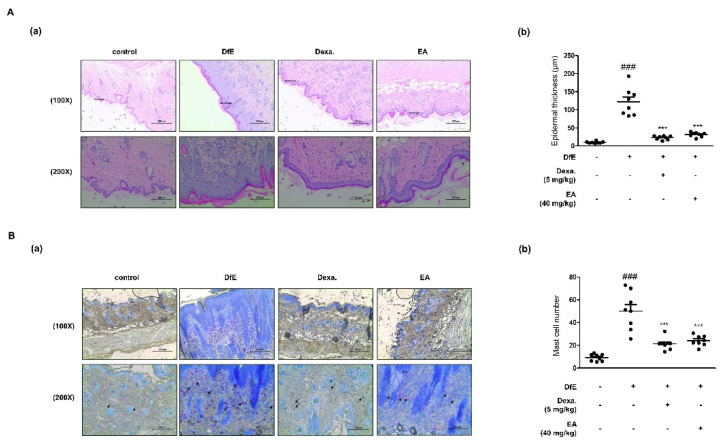
Effects of EA on the histopathological features of DfE-induced AD-like skin lesions in NC/Nga mice (*n* = 8). Dorsal skin was excised, fixed in 10% formaldehyde, embedded in paraffin, and sectioned. The tissue sections were assayed by (**A**) hematoxylin and eosin (H&E) staining and (**B**) toluidine blue staining. ((**A-a**), (**B-a**)) The stained sections were visualized with a microscope at 100× and 200× magnifications. (**A-b**) Epidermal thickness was quantified as the mean of five randomly selected areas per mouse. (**B-b**) Mean of the number of infiltrated mast cells was determined from five randomly selected areas per mouse. Data are expressed as the mean ± standard deviation (SD; *n* = 8). *** *p* < 0.001 versus the only DfE-treated group, ^###^ *p* < 0.001 versus the control group by ANOVA and Dunnett’s post-hoc test.

To figure out whether EA treatment reduced the DfE-induced inflammatory reaction and immune cell infiltration in AD-like skin lesions, histological assays were carried out. Thickened skin and immune cells are deeply involved in cutaneous inflammation [11]. Hematoxylin and eosin (H&E) staining indicated epidermal hyperplasia, edema, and accretion of inflammatory cells in DfE-induced AD-like skin lesions (Figure 5A). DfE induced thickness as compared to the control in both the epidermis and dermis. Both Dexa. and EA suppressed the thickening of the skin. Toluidine blue staining showed that mast cells were piled up in DfE-induced skin lesions (Figure 5B). However, treatment with Dexa. and EA suppressed the inflammatory reactions involving the infiltration of mast cells. Their suppression was significantly more effective as compared to the DfE-treated group.

## 3. Discussion

EA is a phenolic compound found in various fruits and vegetables, especially pomegranates, raspberries, wine, and nuts [16]. It is a dimeric derivative of gallic acid and is sporadically present in food crops, constituting part of ellagitannins. In addition, EA is found in food products conjugated with glycoside moieties [40,41]. Its potent health benefits are diverse, including antiproliferative [42], antioxidative, and anti-inflammatory [22] features, which can be beneficial for treating chronic diseases. However, the effects of EA on the representative chronic skin inflammatory disease AD have not previously been investigated. Thus, we evaluated the effects of EA in human-derived immortalized keratinocytes, HaCaT, and a specialized animal model for AD, NC/Nga mice.

Since AD has a complex pathology, there are a variety of treatment options, including symptomatic therapy, elimination of allergens, and control of immune dysregulation [43]. Among them, topical steroids are the first-line treatment; however, their abuse and misuse frequently cause side effects, such as topical steroid phobia [44]. We also identified a side effect of the steroid Dexa. as weight loss in mice (Figure S1). New treatments for AD regulate inflammatory cytokines, or are antibodies such as dupilumab [7]. In this regard, a natural compound, EA, was investigated for its effects on inflammation in AD.

EA presented a suitable concentration without cell toxicity by 3-(4,5-dimethylthiazol-2-yl)-2,5-diphenyltetrazolium bromide (MTT) assay (data not shown). EA treatment at 250–1000 μM was available for further experiments in HaCaT keratinocytes. For the animal experiment, since EA has an oral toxic dose low for mg/kg/28D for mouse [23], we administrated mice with 40 mg/kg of EA. For in vitro studies, we treated EA at concentrations of 250, 500, and 1000 μM. When keratinocytes are stimulated with pro-inflammatory cytokines TNF-α/IFN-γ, they play a pivotal role in the production of pro-inflammatory cytokines, which affect T lymphocyte differentiation and enlistment of leukocytes to the skin in chronic inflammatory diseases [45]. Therefore, we found increased production and mRNA expression of representative pro-inflammatory mediators IL-6 and TNF-α (Figure 1A). As a primary activator of Th2 cells, IL-6 leads to the secretion of IL-4 and IL-13, promoting the Th2 immune response, which is considered pivotal in the pathogenesis of allergy [46]. With the regulation of secretion of IL-6 under EA treatment, we figured out the mRNA expressions of Th2 chemokines (Figure 1B). Though we did not investigate the effect of EA on Th2 cytokines including IL-4, IL-5, or IL-13 in the current study, we could assume the involvement of EA in regulating Th2-related responses, which is for further study. Since one of primary new treatments for AD is dupilumab, anti-IL-4 receptor α antibody in clinical trials, it would be valuable to investigate the Th2-related immune system for chronic inflammatory diseases [14].

MAPKKs and MAPKs are generally involved in the inflammatory responses, and we found an influence of EA on the cascades, specifically in MEK1/2-ERK and SEK1/MKK4-JNK, in keratinocytes (Figure 2A) [28]. As AD is a Th2-governing disease, TSLP supports Th2 differentiation [47]. When the murine TSLP receptor signals through STAT3 and STAT5/Tec, an Src-type kinase, the human TSLP receptor, activates STAT1, STAT3, STAT4/JAK1, and JAK2 [48]. We studied the effects of EA on STAT1 and STAT3 with protein expression and translocation (Figure 3). In addition, we determined periostin protein expression in keratinocytes under EA. Periostin plays a crucial role in the amplification and persistence of allergic inflammation as an extracellular matrix protein [49]. In the capacity of a matricellular protein, periostin acts on epidermal hyperplasia, which is common in AD [32]. The involvement of periostin in inflammation and AD is assumed to be in the relationship between the MAPKs and STATs in keratinocytes (Figure 2 and Figure 3). The detailed underlying mechanism remains to be investigated further.

Based on the in vitro results of EA, we aimed to confirm the effect on the AD animal model. We used enzyme allergens in the house dust mite DfE for the induction of AD-like skin lesions in NC/Nga mice, which is a well-known animal model of AD [36]. After the first week of induction with sodium dodecyl sulphate (SDS) and DfE, we administrated Dexa. or EA. As a result, the mouse dorsal skin barriers were disrupted with AD symptoms, such as pruritus, erosion, edema, and dryness (Figure 4). Through euthanasia, we confirmed the dryness with TEWL and systemic immunological condition with serum total IgE, TNF-α, and IL-6 production levels. AD-like skin lesions were characterized by the disrupted skin barrier, loss of epidermal water, and elevated IgE levels. To determine the systemic immune activity in AD, we compared the size and weight of immune organs, such as the spleen and lymph nodes (Figure S2). DfE led to a larger size of the immune organs as compared to the smaller size in the EA-treated group under the AD-like condition. In addition, skin-specific lesions and immune responses indicated the effects of EA on the DfE-induced AD animal model with epidermal hyperplasia and mast cell infiltration (Figure 5). These results indicate that EA is effective to alleviate AD-like inflammation.

Results of this study demonstrate that EA alleviates inflammatory responses in AD-like skin lesions in keratinocytes via the suppression the pro-inflammatory mediator expressions underlying MAPK- and STAT-related pathways. Besides, it is known that these findings suggest that EA may be a potent therapeutic agent for the prevention or treatment for skin inflammation such as AD.

## 4. Materials and Methods

### 4.1. Chemicals and Reagents

Dulbecco’s modified Eagle’s medium (DMEM), fetal bovine serum (FBS), penicillin, and streptomycin were obtained from Life Technologies Inc. (Grand Island, NY, USA). Dimethyl sulfoxide (DMSO) was purchased from Junsei Chemical Co., Ltd. (Tokyo, Japan) or Sigma aldrich (St.Louis, MO, USA). We obtained dexamethasone from Merk & Co., Inc. (Kenilworth, NJ, USA) via Sigma Aldrich, Inc. The enzyme immunoassay (EIA) kits for TNF-α, IL-6, and IgE were obtained from BD OptEIA^TM^ (San Jose, CA, USA). SYBR Premix Ex Taq was purchased from TaKaRa (Shiga, Japan). TNF-α, IL-6, TSLP, RANTES, MDC, TARC, and GAPDH oligonucleotide primers were purchased from Bioneer (Daejeon, Chungbuk, Korea). Primary antibodies against p-SEK1/MKK4, SEK1/MKK4, p-ERK, ERK, p-JNK, JNK, p-STAT1 (Ser727), p-STAT1 (Tyr701), STAT1, p-STAT3 (Ser727), p-STAT3 (Tyr701), p-JAK2, and JAK2 were obtained from Cell Signaling Technology (Danvers, MA, USA). Primary antibodies against p-MEK1/2, MEK1/2, p-Akt, Akt, periostin, STAT3, and β-actin as well as peroxidase-conjugated secondary antibodies were obtained from Santa Cruz Biotechnology, Inc. (Santa Cruz, CA, USA).

### 4.2. Preparation of EA

EA was purchased from Cayman Chemical (Ann Arbor, MI, USA) (Cat. No. 10569). We used EA in a concentration of 250, 500, and 1000 μM for in vitro assays and 40 mg/kg for the in vivo assay, respectively. EA was diluted in DMSO (Sigma aldrich, MO, USA) and 10% DMSO for HaCaT cells and NC/Nga mice, respectively.

### 4.3. Cell Culture and Treatment

Human, adult, low calcium, and high temperature (HaCaT) keratinocytes were kindly provided by Prof. Kyung-Tae Lee (Kyung Hee University, Korea) and cultured at 37 °C in DMEM supplemented with 10% FBS, penicillin (100 U/mL), and streptomycin (100 μg /mL) in a humidified atmosphere with 5% CO_2_ every 48–72 h. HaCaT keratinocytes were seeded at a density of 1 × 10^5^ cells/mL. Cells were treated with EA at concentrations of 250, 500, and 1000 μM. They were then stimulated with a TNF-α and IFN-γ mixture (10 ng/mL, each) for the indicated time.

### 4.4. Animals and Treatment

NC/Nga female mice (18–23 g body weight, 6 weeks old) were obtained from Daehan Biolink Co. (Daejeon, Korea). Animals were kept under standard conditions according to the guidelines adopted and promulgated by Sangji University in accordance with the requirements of the National Institutes of Health. Prior to the experiments, the Institutional Animal Care and Use Committee (IACUC) of Sangji University approved all the experimental protocols (IACUC animal approval protocol No. 2018-24; approved on 30 October 2018). Mice were housed (eight mice/cage), acclimatized to the animal room, and fed standard laboratory chow. They were maintained under constant conditions of a 12 h dark/light cycle, temperature of 20 ± 5 °C, and humidity of 40–60%. After acclimatization for 1 week, the mice were randomly divided into four groups. To induce AD-like skin lesions, the back skin was topically treated with 100 mg/mouse crude extract of DfE (Biostir^®^ AD; Biostir, Hyogo, Japan). Mite antigen was applied twice weekly for 4 weeks (Figure 4A). The skin barrier was disrupted with 150 μL of 4% SDS following the application of DfE ointment for 3 h. With the disruption application, Dexa. (5 mg/kg, per os) and EA (40 mg/kg, intraperitoneal administration) were administered after 4 h of DfE treatment. Mice were divided into four groups as follows—(1) the control group with no SDS or DfE application; (2) the DfE-treated group with DfE application (DfE-induced AD-like lesions); (3) the positive control group with DfE treatment (Dexa., 5 mg/kg); and (4) the EA-administered group with DfE treatment (EA, 40 mg/kg). Mice were euthanized by cervical dislocation 4 weeks after the first application of DfE, and blood was collected from them. Dorsal skin tissues were isolated for histological examination.

### 4.5. Evaluation of Dermatitis Severity

The severity of dermatitis was scored using the Merkmal of symptom score, as described by Yamamoto et al. [37]. The progress of dermatitis was evaluated once a week. The aggravation of edema, scarring/dryness, erythema/hemorrhage, and excoriation/erosion was scored as follows—0, none; 1, mild (severity < 20%); 2, moderate (severity = 20–60%); and 3, severe (severity > 60%). The total of the individual scores was used as the dermatitis score.

### 4.6. Transepidermal Water Loss

As a healthy skin barrier protects hydrated skin from water loss, the epidermal water loss was evaluated with gpskin barrier light (gpower, Seoul, Korea). On the final day of the experiment, TEWL was measured on the dorsal skin with the machine when the mice were alive right before euthanasia.

### 4.7. Histological Analysis of Skin Lesions

Skin samples from the dorsal area were isolated after euthanasia. The samples were fixed in 10% buffered formalin and then embedded in paraffin. Next, they were sectioned into 8-μM slices and stained with hematoxylin and eosin (H & E). Pathological changes, such as hyperkeratosis, dermal edema, epidermal and dermal hyperplasia, vesicular formation, parakeratosis, and inflammation were evaluated. Selected sections were stained with toluidine blue to assess mast cell infiltration. The average count of mast cells in each specimen was used to determine the mast cell density/mM^2^. Images were captured under an optical microscope (Leica DFC 295, Wetzlar, Germany).

### 4.8. mRNA Extraction and Quantification

Total RNA was isolated from the back skin tissue and HaCaT cells using the Easy Blue RNA extraction kit, according to the manufacturer’s instructions. A cDNA reverse transcription kit (Life Technologies, Grand Island, NY, USA) was used, and reverse transcription was conducted with a GeneAmp PCR System 9700 (Applied Biosystems, Foster City, CA, USA) with SYBR premix Ex Taq. The synthesized cDNAs had a size of 200 bp. StepOnePlus^®^ Real-Time PCR system (Applied Biosystems) was used for amplification with SYBR Green PCR Master Mix. Expression data were calculated from the cycle threshold (Ct) value using the ΔCt method of quantification (2^−ΔΔCt^ method). GAPDH was used for normalization. The primer information for qRT-PCR is listed in Table 1.

### 4.9. Western Blot Analysis

Protein extracts from dorsal skin and HaCaT cells were prepared using PRO-PREP™ protein extraction solution (Intron Biotechnology, Seoul, Korea) and homogenized at 4 °C. Tissue debris was removed by microcentrifugation after immediate freezing of the supernatant. The protein concentration was determined using the Bio-Rad protein assay reagent, according to the manufacturer’s instructions. After 8–12% SDS-polyacrylamide gel electrophoresis, protein from each group was electroblotted onto a polyvinylidene difluoride membrane. The immunoblot was incubated with a blocking solution (2.5–5% skim milk) for 30 min at room temperature and incubated overnight with a primary antibody (dilution, 1:1000 in Tween 20/Tris-buffered saline (TBST)) at 4 °C. After washing thrice with TBST, blots were incubated with a horseradish peroxidase-conjugated secondary antibody (dilution, 1:2000) for 2 h at room temperature. Blots were washed again thrice with TBST and then visualized using enhanced chemiluminescence (GE Healthcare Life Sciences, Chalfont, UK) and X-ray film (Agfa, Belgium). Densitometric analysis was performed using Bio-Rad Quantity One software.

### 4.10. Cytokine Analysis

Blood was collected from the orbital sinus of each mouse at the end of the experiment. Serum was obtained by centrifugation at 1700× *g* for 30 min and kept at −70 °C until analysis. The serum levels of total TNF-α, IL-6, and IgE were measured using mouse TNF-α, IL-6, and IgE ELISA kits (BD OptEIA^TM^, BD Science, CA, USA), according to the manufacturer’s instructions. Culture media were obtained approximately 24 h after treatment with EA and stored at −70 °C. The production levels of TNF-α and IL-6 were assessed using EIA kits for humans (BD OptEIA^TM^, BD Science, San Jose, CA, USA) according to the manufacturer’s instructions.

### 4.11. Immunofluorescence Assay

HaCaT keratinocytes were cultured directly on a chamber slide (Lab-Tek II chamber slide #154526, four-well) at a density of 0.5 × 10^5^ cells/mL for 1 h to detect p-STAT1 (Tyr701) localization. After stimulation with TNF-α/IFN-γ in the presence or absence of EA, the cells were fixed with 100% methanol for 30 min at room temperature and blocked with 10% normal goat serum. The cells were incubated overnight with specific primary antibodies in 10% blocking solution. After washing the primary antibodies with 0.3% Triton X in phosphate-buffered saline for 30 min, Alexa Fluor 488 goat anti-rabbit IgG (Invitrogen, Carlsbad, CA, USA) was applied and incubated for 1 h. Cells were mounted with mounting medium containing DAPI (Life Technologies, Carlsbad, CA, USA) and observed under optical microscopy (ECLIPSE Ni-U; Nikon, Tokyo, Japan).

### 4.12. Statistical Analysis

Data were expressed as the mean ± SEM of three experiments (in vitro) and as the mean ± SD of animal experiment. Comparisons among groups were carried out using one-way ANOVA followed by Dunnett’s post-hoc test served in GraphPad Prisem5 (GraphPad Software, San Diego, CA, USA). P-values of less than 0.05 were considered statistically significant.

## 5. Conclusions

In conclusion, the present study demonstrated that EA suppressed the inflammatory responses in HaCaT keratinocytes and alleviated AD-like skin lesions in NC/Nga mice. We assumed that these inhibitory effects of EA on inflammatory responses were mediated by MAPK and STAT pathways.

## Figures and Tables

**Table 1:** Primer sequences.

Gene Name	Forward Primers (5′-3′)	Reverse Primers (5′-3′)
TNF-α (h)	CGCTCCCCAAGAAGACAG	AGAGGCTGAGGAACAAGCAC
IL-6 (h)	ATTCCGGGAACGAAAGAGAA	TCTTCTCCTGGGGGTACTGG
TSLP (h)	CAGGCTATTCGGAAACTCAGA	GTAATTGTGACACTTGTTCCAGAC
RANTES (h)	CCGCGGCAGCCCTCGCTGTCATCC	CATCTCCAAAGAGTTGATGTACTCC
MDC (h)	AGGACAGAGCATGGATCGCCTACAGA	TAATGGCAGGGAGGTAGGGCTCCTGA
TARC (h)	CTTCTCTGCAGCACATCC	AAGACCTCTCAAGGCTTTG
GAPDH (h)	TGCACCACCACCTGCTTAGC	GGCATGGACTGTGGTCATGAG

## Data Availability

The analyzed data sets generated during current study are available from the corresponding author on reasonable request.

## Supplementary Materials

The following are available online at https://www.mdpi.com/1422-0067/22/3/1277/s1.

## Abbreviations

AD	atopic dermatitis
EA	ellagic acid
DfE	*Dermatophagoides farinae* extract
MAPK	mitogen-activated protein kinase
STAT	signal transducer and activator of transcription
