# Supplementary material for: Anti-Inflammatory Effects of Ellagic Acid on Keratinocytes via MAPK and STAT Pathways
Source: Int J Mol Sci. 2021 Jan 28;22(3):1277. doi: 10.3390/ijms22031277 (PMC7865693; doi:10.3390/ijms22031277)
Supplement: Supplementary file 1 [file ijms-22-01277-s001.pdf]

S1

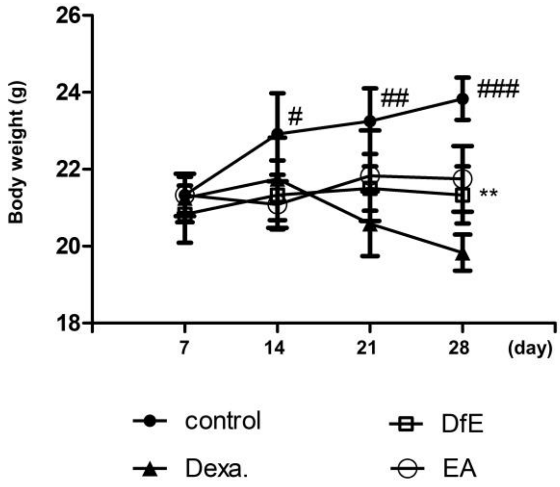

Figure S1. Effect of EA on body weight in DfE-induced AD-NC/Nga mice (n = 8). Body weights were measured every week. Each group consisted of six mice. **p < 0.01 *versus* the only DfE-treated group, #p < 0.05, ##p < 0.01, and ###p < 0.001 *versus* the control group by ANOVA and Dunnett's *post-hoc* test.

S2

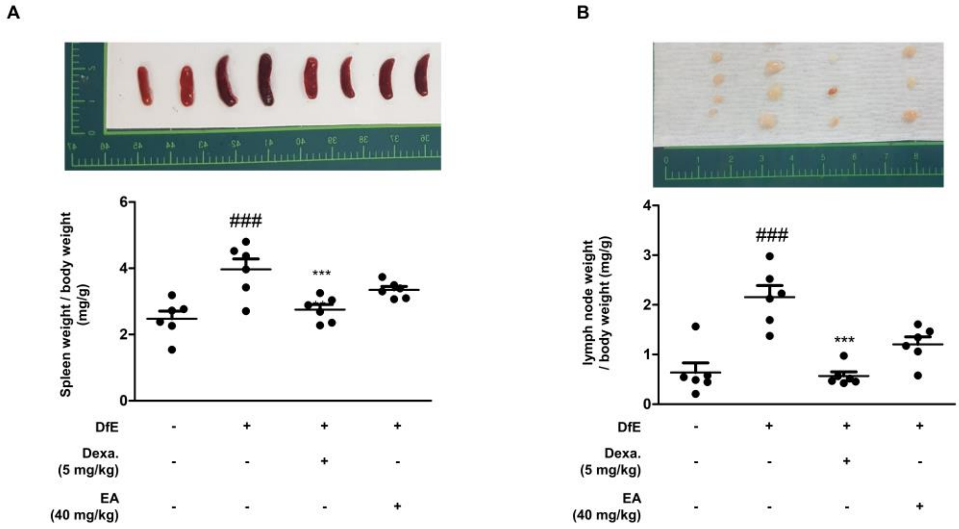

Figure S2. Effect of EA on immune organ weight in DfE-induced AD-NC/Nga mice. (A) The spleen was isolated from each mouse and (B) representative lymph nodes were detached from the groin and axilla (n = 6). ***p < 0.001 *versus* the only DfE-treated group, ###p < 0.001 versus the control group by ANOVA and Dunnett's *post-hoc* test.
